# Hospitalization for musculoskeletal disorders in rheumatoid arthritis patients: a population-based study

**DOI:** 10.1186/s12891-016-1142-4

**Published:** 2016-07-19

**Authors:** Marina Amaral de Ávila Machado, Sasha Bernatsky, Louis Bessette, Hacene Nedjar, Elham Rahme

**Affiliations:** Research Institute of the McGill University Health Centre, Montreal, QC Canada; College of Medicine, Federal University of Minas Gerais, Belo Horizonte, Minas Gerais Brazil; Department of Medicine, Division of Clinical Epidemiology, McGill University Health Centre, 687 Pine Ave West, V building, Montreal, QC H3A 1A1 Canada; Centre Hospitalier Universitaire de Québec, Laval University, Quebec City, QC Canada

**Keywords:** Rheumatoid arthritis, DMARDs, Anti-TNF, Musculoskeletal conditions

## Abstract

**Background:**

Rheumatoid arthritis (RA) patients failing disease modifying antirheumatic drugs (DMARDs) may undergo anti-Tumor Necrosis Factor (anti-TNF) therapy. Using the Quebec health services administrative databases, we examined the rates of musculoskeletal (MSD)-related hospitalizations among RA patients receiving anti-TNF, DMARDs, and neither of those therapies (non-users).

**Methods:**

Matched cohort analyses were performed separately in 2002–2006 and 2007–2011. In each cohort, DMARD and non-user groups were formed to 3-1 match the anti-TNF users on age, sex, date of RA diagnosis, high-dimensional propensity score and date of the first anti-TNF dispensation (index-date). Non-users did not use DMARDs or anti-TNF drugs during the year before the index-date and in the 90 days post, but used at least one of these medications in the study period.

**Results:**

During 2002–2006, 557 anti-TNF users were matched to 1144 DMARD users and to 656 non-users, compared to 690, 1651, and 532 patients, respectively during 2007–2011. The crude rates of MSD-related hospitalizations in the anti-TNF, DMARD and non-users groups were respectively: 8.2/100, 6.4/100 and 10.5/100 patient-years in 2002–2006, and 6.9/100, 4.8/100, and 8.6/100 patient-years in 2007–2011. In multivariable Cox regression models, the hazard ratios of MSD-related hospitalizations (95 % confidence interval) were: 0.95 (0.60; 1.50) for anti-TNF and 0.69 (0.46; 1.02) for DMARD users, versus non-users in 2002–06, and 0.65 (0.37; 1.14) and 0.40 (0.24; 0.66), respectively in 2007–2011.

**Conclusion:**

The MSD-related hospitalization risk was lower in RA patients using DMARD therapy and similar in those using anti-TNF therapy with or without DMARDs as compared to those not using either of these therapies during the study period.

## Background

Clinical practice guidelines recommend that disease-modifying anti-rheumatic drugs (DMARD) be introduced in rheumatoid arthritis (RA) as soon as possible. Combination therapy with DMARDs (methotrexate +/−hydroxychloroquine +/− sulfasalazine) and/or the addition of biologic agents that target Tumor Necrosis Factor (TNF) is considered in patients who have an inadequate response to DMARD monotherapy. Corticosteroids are also used, to manage flares and suppress symptoms [1]

In Quebec, anti-TNF drugs were listed on the public drug formulary for RA in 2002. Eligibility for an anti-TNF remains active synovitis (eight or more joints), and having failed two DMARDs including methotrexate [2]. As such, anti-TNF drug users would be expected to have more severe RA compared to non-users, with a trend towards more prompt initiation in more recent times [3–5].

Randomized controlled trials (RCT) and observational studies have demonstrated benefits of anti-TNF agents in RA treatment on the basis of both disease activity and joint damage [6, 7]. Some observational studies have considered hospital admissions as an effectiveness indicator showing that anti-TNF therapy may reduce the rate of hospitalization, although the results remain uncertain [8, 9].

We used a high-dimensional propensity score approach [10] with Quebec health services administrative data to compare the rates of musculoskeletal (MSD) –related hospitalizations among RA patients receiving anti-TNF therapy, those receiving DMARDs, and those patients who were receiving neither of those therapies (non-users). We also compared results across calendar time. We hypothesized that MSD-related hospital admission rates were lower in RA patients using anti-TNF agents compared to patients using DMARDS.

## Methods

### Data sources

We used physician and prescription drug claims, hospital discharge data, and demographic records from January 1997 to March 2012 from the provincial health services administrative databases administered by the Régie de l’assurane maladie du Québec (RAMQ). In this Canadian province, coverage for outpatient and inpatient physician services is provided for the entire population (about 7.5 million people). Individuals aged 65 years or older (1,106,428 individuals in 2011; 90 % of that population) and those under 65 years (2,261,786 individuals in 2011; 32 % of that population including those who receive social assistance, 493 212 individuals, and those who do not have collective private drug insurance, 1,768,574 individuals, such as the self-employed), have their prescription drugs covered by the provincial government. Medication dispensations have been demonstrated to be accurately and reliably recorded in the RAMQ prescription claims database [11]. The Quebec hospital discharge abstract database provides information on all hospital admissions including primary and secondary discharge diagnoses [International Classification of Diseases (ICD), 9^th^ revision (ICD-9) codes until April 2006 and 10^th^ revision (ICD-10) codes thereafter] and admission/discharge dates. The databases are linkable by a unique patient identifier. Permission to link the data was obtained from the Provincial Ethics Board, the Commission d’accès à l’information. Approval to conduct the study was obtained from the McGill University Health Centre Ethics Review Board.

### Study design

As anti-TNF users were expected to have failed at least two DMARDs and to have a more severe RA profile in the earlier years of marketing, we conducted our main analyses over two separate time-periods January 2002 - December 2006 and January 2007 - December 2011 and results were compared. To further understand the effect of anti-TNF on the study outcomes, we also ran our analyses over the time-period immediately preceding the introduction of anti-TNF medications on the Quebec drug formulary, January 1998 - December 2001.

We describe the study cohort in 2002–2006. Study cohorts of 1998–2001 and 2007–2011 are similarly constructed. An RA cohort was first constructed including patients 20 years of age and older who in 2002–2006 had two outpatient ICD-9 codes for RA (714.x) at least 30 days and no more than 2 years apart or one diagnosis (principal or secondary) from the hospital abstract database (ICD-9 code 714.x until April 2006 and ICD-10 codes M05.x, M06.x, M08.x, and M09.x afterwards) [12]. Individuals who received at least one dispensed prescription for either a DMARD or anti-TNF during the study period were considered in the study. The anti-TNF agents available in Quebec during 2002–2006 were: infliximab, etanercept, adalimumab. Certolizumab, rituximab and golimumab became available in the period 2007–2011 and were also included. The DMARDs included in the study were: chloroquine, hydroxychloroquine, cyclophosphamide, cyclosporine, methotrexate, minocycline, penicillamine, auranofine, sulfasalazine, leflunomide, aurothioglucose, aurothiomalate. Other non-anti-TNF biologic drugs were not included in this study because of the very small number of users.

### Study cohorts

Each study cohort consisted of three groups, anti-TNF, DMARDs and non-users. Patients in the RA cohort dispensed an anti-TNF in 2002–2006 were identified at the first dispensation date (index date). Those covered by the provincial drug plan for at least 1 year prior and 3 months post the index date were included and formed the anti-TNF group (anti-TNF naïve in the previous year). Two other groups, the DMARD and non-user groups, were formed to match the anti-TNF group on age (±2 years), sex, date of RA diagnosis (±90 days) and high-dimensional propensity score (±0.05) [10]. To form the DMARD group, for each individual in the anti-TNF group, three anti-TNF naïve individuals (for at least 1 year before and 90 days after the date of anti-TNF dispensing) who used a DMARD within 90 days of the index date of the anti-TNF individual were selected at random from those eligible at the date of the DMARD use (index date; for those who used more than one DMARD within the 90-day-period, the closest date to the anti-TNF index date was chosen). The non-user group was constructed similarly. For each individual in the anti-TNF group, three anti-TNF and DMARD naïve individuals were selected at random to match the anti-TNF individual on age, sex, date of RA and high-dimensional propensity score as described above. The index date of the anti-TNF individual was assigned as the index date of the non-user individuals. Of note, patients in the non-user group did not use an anti-TNF or a DMARD for at least 1 year before and 90 days after the index date, but have used at least one of these drugs at another time during the study period as described above. The non-users could be under therapy with other drugs, such as corticosteroids and nonsteroidal anti-inflammatory drugs (NSAIDs). The high-dimensional propensity score has been proposed to adjust for indication and confounding biases caused by missing or misclassified information. In this study, the high-dimensional propensity score (probability of receiving an anti-TNF) was calculated in a multivariate logistic regression model adjusted for a large number of covariates (500), as suggested in the algorithm, assessed based on all diagnoses, procedures, services and drug-dispensations recorded in the databases and selected according to their potential to bias the exposure-outcome relationship under study [10]. A high–dimensional propensity score algorithm is available as downloadable SAS software files from the Brigham and Women’s Hospital [13]. All included patients were required to be alive for at least 90 days past their index date and not to have had any MSD-related hospitalization in the prior year.

All study patients were followed from index date until the first date of death, end of drug coverage, switch/discontinuation of their index treatment or a maximum of 1 year. Discontinuation of treatment was defined as at least 90 days without the treatment and treatment switch was a switch between the three exposure groups (a switch between two DMARDs or two anti-TNFs was not considered to be a treatment switch).

### Outcomes

The first hospitalization with a principal diagnosis for any MSD reason, ICD-9 and 10 codes included in the chapter XIII - diseases of the MSD system and connective tissue, during follow-up was the principal outcome (Appendix 1 and Appendix 2– diagnoses found in the study).

### Patient baseline characteristics

Patient characteristics assessed at index date included: age, sex, type of insurance plan (based on patient eligibility for premium subsidies; low income patients were those receiving premium or partial subsidies), region of residence (urban or rural), visits to a rheumatologist in the prior year, comorbidity (cancer, ischemic heart disease, congestive heart failure (CHF), peptic ulcer disease, cerebrovascular disease (CVD), atrial fibrillation, and hematologic disorders), medication used in the prior year [corticosteroids, gastroprotective agents (proton pump inhibitors, misoprostol and histamine-2 receptor blocker), serotonin reuptake inhibitors (SSRI), anticoagulants, antidiabetics, antihypertensives and NSAIDs]. These factors were chosen because of their potential association with the choice of RA treatment and the outcome (MSD-related hospitalizations). In addition, our data included an index of socioeconomic status (SES), with sub-indices of social and material deprivation, that was developed by the Institut National de Santé Publique du Québec on the basis of census enumeration area data on education level, employment/population ratio, and average income [14, 15].

### Secondary analyses

In secondary analyses, patient selection, variable assessment and statistical analyses described above were repeated for patients on DMARDs and patients on neither drug, in the three periods (1998–2001, 2002–2006 and 2007–2011). The DMARD group in this analysis consisted of all patients who used a DMARD, but had never been on an anti-TNF prior to the DMARD use, as opposed to the previous analysis where the DMARD group was selected to match the anti-TNF group.

### Statistical analyses

The following analyses were conducted separately in each of the study periods. Descriptive analyses [mean and standard deviation (SD) or proportion] were used as appropriate to report baseline patient characteristics by treatment group. Polytomous logistic regression models were used to compare patient baseline characteristics between the three treatment groups. The crude rates/100 patient-years (py) of MSD-related hospitalizations were assessed. Kaplan Meier curve displayed time to the first MSD-related hospitalization in the three treatment groups. Multivariable Cox proportional hazard models were used to compare the hazard ratios of MSD-related hospitalizations between treatment groups adjusting for patient baseline characteristics.

All statistical analyses described above were repeated in secondary analyses to compare MSD-related hospitalizations in the DMARD users versus non-users in the three time-periods. All statistical analyses were performed using SAS version 9.4 for UNIX (SAS Institute Inc., Cary, NC).

## Results

In total, 10,418 RA individuals were in the 2002–2006 cohort, and 15,936 in the 2007–2011 cohort (data not shown). Among these, 557 used anti-TNF, 1144 were matched DMARD users and 656 matched non-users in 2002–2006; while, 690 used anti-TNF, 1651 were matched DMARD users and 532 matched non-users in 2007–2011 (Table 1). Among non-users, 81 % used corticosteroid and/or NSAID in the follow-up during 2002–2006 compared to 74 % during 2007–2011 (Appendix 3).

**Table 1:** Baseline characteristics of individuals with rheumatoid arthritis in Quebec in 2002–2006 and 2007–2011

Variables	2002–2006			2007–2011		
	Anti-TNF (557)	DMARDs (1144)	Non-users (656)	Anti-TNF (690)	DMARDs (1651)	Non-users (532)
Demographics						
Age (mean (±SD)) years	63.0(11.5)	64.2 (10.8)	65.1 (10.6)	65.2 (10.5)	66.2 (9.7)	68.4 (9.3)
Sex (female *N* (%))	426 (76.5)	921 (80.5)	562 (85.7)	517 (74.9)	1281 (77.6)	426 (80.1)
Residence (urban *N* (%))	428 (76.8)	899 (78.6)	526 (80.2)	531 (77.0)	1316 (79.7)	425 (79.9)
Higher income^a^	344 (61.8)	691 (60.4)	379 (57.8)	417 (60.4)	980 (59.4)	339 (63.7)
Socioeconomic status *N* (%)						
Social quintile 0	58 (10.4)	90 (7.9)	48 (7.3)	49 (7.1)	100 (6.1)	25 (4.7)
Social quintile 1	106 (19.0)	211 (18.4)	88 (13.4)	113 (16.4)	285 (17.3)	74 (13.9)
Social quintile 2–3	192 (34.5)	392 (34.3)	238 (36.3)	251 (36.5)	618 (37.4)	218 (41.0)
Social quintile 4–5	201 (36.1)	451 (39.4)	282 (43.0)	276 (40.0)	648 (39.2)	215 (40.4)
Use of health services in prior year *N* (%)						
Visit to rheumatologist	490 (88.0)	946 (82.7)	480 (73.2)	608 (88.1)	1365 (82.7)	338 (63.5)
Comorbidity in prior year *N* (%)						
Hematologic disorders	75 (13.5)	112 (9.8)	69 (10.5)	72 (10.4)	182 (11.0)	69 (13.0)
Heart failure	16 (2.9)	29 (2.5)	9 (1.4)	19 (2.8)	38 (2.3)	23 (4.3)
Cerebrovascular disease	21 (3.8)	23 (2.0)	19 (2.9)	10 (1.4)	39 (2.4)	22 (4.1)
Atrial fibrillation	14 (2.5)	24 (2.1)	13 (2.0)	23 (3.3)	54 (3.3)	18 (3.4)
Ischemic heart disease	79 (14.2)	138 (12.1)	76 (11.6)	65 (9.4)	186 (11.3)	56 (10.5)
Peptic ulcer disease	7 (1.3)	10 (0.9)	5 (0.8)	1 (0.1)	9 (0.5)	3 (0.6)
Cancer	39 (7.0)	102 (8.9)	51 (7.8)	69 (10.0)	172 (10.4)	64 (12.0)
Medication use in prior year *N* (%)						
NSAIDs	430 (77.2)	843 (73.7)	452 (68.9)	435 (63.0)	980 (59.4)	248 (46.6)
Serotonin reuptake inhibitors	57 (10.2)	107 (9.4)	62 (9.5)	71 (10.3)	164 (9.9)	532 (9.6)
Gastroprotective agents	322 (57.8)	664 (58.0)	345 (52.6)	463 (67.1)	970 (58.8)	328 (61.7)
Antidiabetics	63 (11.3)	115 (10.1)	51 (7.8)	81 (11.7)	170 (10.3)	77 (14.5)
Corticosteroid	414 (74.3)	757 (66.2)	400 (61.0)	500 (72.5)	928 (56.2)	306 (57.5)
Anticoagulants	27 (4.8)	49 (4.3)	31 (4.7)	32 (4.6)	106 (6.4)	34 (6.4)
Antihypertensives	294 (52.8)	587 (51.3)	305 (46.5)	414 (60.0)	944 (57.2)	335 (63.0)

^a^Those who do not receive any guaranteed income supplement

### Patient baseline characteristics

Matching by high-dimensional propensity score, age and sex, removed most differences in baseline patient characteristics between the treatment groups except those related directly to the treatment choice such as prior corticosteroid and NSAID use, prior visits to rheumatologists and socioeconomic status (Table 2). In 2002–2006, patients in the anti-TNF group and those in the DMARD group had higher SES compared to non-users and were more likely than non-users to have taken corticosteroids and NSAIDs and to have visited a rheumatologist in the previous year. In 2007–2011, patients in the anti-TNF group were more likely than non-users to live in rural areas, to have received partial or total subsidies, to have used corticosteroid and NSAIDs and to have seen a rheumatologist in the previous year. They were also less likely to have CVD. In 2007–2011, patients in the DMARD group were more likely than non-users to have received partial or total subsidies, to have taken NSAIDs and visited a rheumatologist in the previous year. They were also less likely to have CHF and to have been using antidiabetics.

**Table 2:** Patient characteristics associated with anti-TNF and DMARD use: logistic regression model

Variables	2002–2006		2007–2011	
	OR (95 % CI)		OR (95 % CI)	
	Anti-TNF vs Non-users	DMARDs vs Non-users	Anti-TNF vs Non-users	DMARDs vs Non-users
Demographics				
Residence (rural vs urbain)	1.21 (0.88, 1.66)	1.05 (0.81, 1.38)	1.43 (1.05, 1.96)	1.15 (0.87, 1.51)
Higher income	1.14 (0.90, 1.46)	1.02 (0.83, 1.26)	0.76 (0.59, 0.98)	0.70 (0.56, 0.87)
Socioeconomic status				
Social quintile 0	1.02 (0.62, 1.68)	0.75 (0.48, 1.17)	1.33 (0.73, 2.41)	1.00 (0.59, 1.71)
Social quintile 2–3 versus 1	0.69 (0.48, 0.98)	0.69 (0.51, 0.93)	0.71 (0.49, 1.01)	0.72 (0.53, 0.98)
Social quintile 4–5 versus 1	0.61 (0.43, 0.88)	0.66 (0.49, 0.90)	0.82 (0.57, 1.18)	0.74 (0.54, 1.01)
Use of health services in prior year				
Visit to rheumatologist	2.73 (1.97, 3.79)	1.74 (1.36, 2.22)	4.41 (3.27, 5.95)	2.86 (2.29, 3.60)
Comorbidity in prior year				
Hematologic disorders	1.32 (0.90, 1.94)	0.92 (0.66, 1.28)	0.88 (0.60, 1.30)	0.96 (0.69, 1.32)
Heart failure	1.97 (0.84, 4.61)	1.95 (0.89, 4.29)	0.82 (0.41, 1.63)	0.52 (0.28, 0.94)
Cerebrovascular disease	1.43 (0.74, 2.75)	0.75 (0.39, 1.42)	0.38 (0.16, 0.86)	0.63 (0.35, 1.14)
Ischemic heart disease	1.06 (0.72, 1.55)	0.90 (0.64, 1.26)	0.97 (0.63, 1.50)	1.33 (0.93, 1.91)
Cancer	0.86 (0.55, 1.35)	1.17 (0.81, 1.69)	0.82 (0.56, 1.20)	0.88 (0.64, 1.22)
Medication use in prior year				
NSAIDs	1.59 (1.21, 2.09)	1.26 (1.02, 1.58)	1.87 (1.46, 2.40)	1.66 (1.35, 2.05)
Serotonin reuptake inhibitors	0.93 (0.62, 1.39)	0.90 (0.65, 1.26)	1.08 (0.72, 1.63)	1.12 (0.79, 1.60)
Gastroprotective agents	1.04 (0.81, 1.33)	1.18 (0.95, 1.45)	1.09 (0.84, 1.42)	0.85 (0.68, 1.06)
Antidiabetics	1.39 (0.93, 2.08)	1.28 (0.89, 1.83)	0.76 (0.52, 1.09)	0.71 (0.53, 0.97)
Corticosteroid	1.87 (1.44, 2.43)	1.22 (0.99, 1.50)	1.92 (1.49, 2.47)	0.96 (0.78, 1.18)
Anticoagulants	0.90 (0.50, 1.62)	0.84 (0.52, 1.37)	0.98 (0.56, 1.71)	1.39 (0.89, 2.19)
Antihypertensives	1.21 (0.94, 1.57)	1.20 (0.97, 1.48)	0.93 (0.72, 1.21)	0.85 (0.68, 1.06)

### Hospitalizations for MSD-related events

**Fig. 1:**
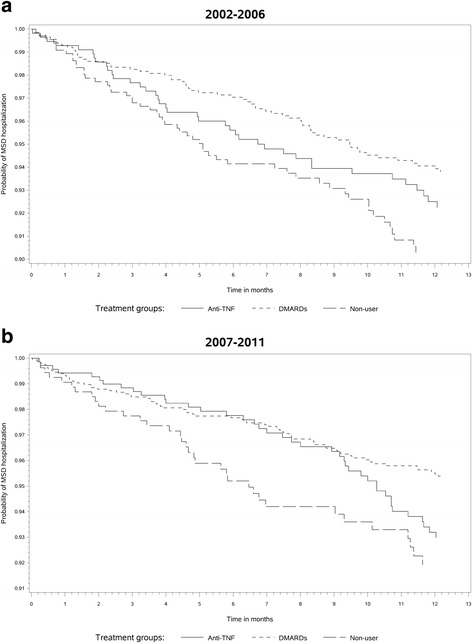
Time to admission to the first hospitalization for a musculoskeletal condition: Kaplan Meier curves

The total number of patients who had MSD-related hospitalisations in 2002–2006 and 2007–2011 are displayed in Table 3. In 2002–2006, among the anti-TNF group, 39 individuals were hospitalized (crude rate 8.2/100 py), compared to 63 patients (6.4/100 py) among the DMARDs group and 53 patients (10.5/100 py) among the non-users group. While, in 2007–2011, 40 patients among the anti-TNF group were hospitalized for MSD-related events (6.8/100 py), compared to 70 patients (4.8/100 py) among the DMARDs group and 35 patients (8.6/100 py) among the non-user group. Figure 1 displays the Kaplan Meier curves of time to first MSD-related hospitalizations in the three treatment groups. In stratified multivariate Cox proportional hazard models, ischemic heart disease increased the risk of MSD-related hospitalizations in both periods. Use of antihypertensive agents in the previous year was negatively associated with MSD-related hospitalization in 2002–2006. Prior visits to rheumatologists were associated with a decreased risk of MSD-related hospitalizations in 2007–2011 (Table 4). In these models, the risk of MSD-related hospitalizations did not differ between anti-TNF users and non-users (0.95, 0.60, 1.50) in 2002–2006. The risk in that period tended to be lower in DMARD users 0.69 (0.46, 1.02). In 2007–2011, the risk in anti-TNF users tended to be lower than that in non-users, although statistical significance was not reached (0.65; 0.37, 1.14). The risk in the DMARDs group was lower than that in non-users during this period (0.40; 0.24, 0.66).

**Table 3:** Exposure duration, unadjusted rates and adjusted rate ratios (Cox proportional hazard models) of musculoskeletal hospitalizations in 2002–2006 and 2007–2011

	Exposure duration (days)		Hospitalization for musculoskeletal conditions	
	Total	Median (interquartile range)	Number (unadjusted rate per 100 patient-yrs)	Hazard ratio (95 % CI)
2002–2006				
Anti-TNF	174 128	365.0 (79.0)	39 (8.18)	0.95 (0.60, 1.50)
DMARDs	358 954	365.0 (74.0)	63 (6.41)	0.69 (0.46, 1.02)
Non-user	184 986	349.0 (173.0)	53 (10.46)	1.00 (Reference)
2007–2011				
Anti-TNF	213 189	365.0 (102.0)	40 (6.85)	0.65 (0.37, 1.14)
DMARDs	527 820	635.0 (54.0)	70 (4.84)	0.40 (0.24, 0.66)
Non-user	148 381	349.5 (181.0)	35 (8.61)	1.00 (Reference)

*CI* confidence interval

**Table 4:** Patients characteristics associated with hospitalization for musculoskeletal conditions: Cox proportional hazard models adjusted for treatment group at baseline

Demographics	2002–2006	2007–2011
Use of health services in prior year		
Visit to Rheumatologist	-	0.51 (0.30, 0.86)
Comorbidity in prior year		
Ischemic heart disease	2.34 (1.32, 4.13)	2.69 (1.38, 5.22)
Medication use in prior year		
Antihypertensives	0.71 (0.47, 1.08)	-

Also adjusted for patient characteristics listed in Table 2. Only significant associations are reported in this table

### Secondary analyses

Repeating the selection of cohorts and analyses among DMARDs users and non-users in 1998–2001, 2002–2006 and 2007–2011 revealed that in total, 3844 individuals used DMARDs and 7356 were matched non-users in 1998–2001; compared to 5978 DMARDs users and 11,439 matched non-users in 2002–2006; and 8260 DMARDs users and 15,361 matched non-users in 2007–2011. The total numbers of patients who were hospitalized for MSD-related events in the three periods are displayed in Table 5 by treatment group. In 1998–2001, the numbers of hospitalized patients (crude rate/100 py) were 229 (6.7/100 py) in the DMARD and 362 (6.6/100 py) in the non-user groups. While, in 2002–2006, they were 289 (5.4/100 py) in the DMARD and 614 (6.9/100 py) in the non-user groups; and in 2007–2011, they were 344 (4.5/100 py) in the DMARD and 797 (7.3/100 py) in the non-users groups. In multivariable Cox proportional Hazard models, the hazard ratios of MSD-related hospitalizations in the DMARD versus non-users groups were 1.16 (0.95, 1.41) in 1998–2001, 0.86 (0.73, 1.01) in 2002–2006 and 0.71 (0.60, 0.84) in 2007–2011.

**Table 5:** Secondary analyses comparing Hospitalizations for musculoskeletal conditions among DMARDs users and non-users before and after the introduction of anti-TNF drugs to the Quebec market

	Exposure duration (days)		Number (Unadjusted rate per 100 patient-yrs)	Hazard ratio (95 % CI)
	Total	Median (Quartile range)		
1998–2001				
DMARDs	1,243,393	365.0 (12.0)	229 (6.72)	1.16 (0.95, 1.41)
Non-users	1,998,164	317.0 (193.0)	362 (6.61)	1.00 (Reference)
2002–2006				
DMARDs	1,949,824	365.0 (0.0)	289 (5.41)	0.86 (0.73, 1.01)
Non-users	3,270,739	365.0 (169.0)	614 (6.85)	1.00 (Reference)
2007–2011				
DMARDs	2,785,950	365.0 (0.0)	344 (4.51)	0.71 (0.60, 0.84)
Non-users	3,970,012	296.0 (224.0)	797 (7.33)	1.00 (Reference)

*CI* confidence interval

## Discussion

Our results suggests that in RA patients, the risks of MSD-related hospitalizations were similarly likely for those using anti-TNF therapy compared to non-users. The risk seemed higher in the first 5 years after the introduction of anti-TNF drugs to the market compared to the following 7–11 years. In DMARDs users that matched the anti-TNF users, MSD-related hospitalizations were less likely than in non-users in both periods, although results reached statistical significance in the second period. Analyses of all DMARD users revealed a similar risk among DMARD users compared to non-users in the period preceding the introduction of the anti-TNF to the market and a decreasing trend showing a lower risk among DMARD users in the following two periods. The apparently lower risk found in DMARD versus anti-TNF users is not surprising as anti-TNFs can only be prescribed in Quebec when DMARD therapy has failed. However, the higher risk of MSD-related hospitalizations among non-users is somehow concerning. The reasons for not using DMARDs or anti-TNF therapy during the study period among non-users was not known in our study. Further examination of the data revealed that the majority of non-users (81 % in 2002–2006 and 74 % in 2007–2011) used corticosteroid and/or NSAID in the follow-up. However, this alone cannot explain the higher hospitalization rate observed among non-users since the anti-TNF and DMARD groups also used these mediations in follow-up in similar to slightly higher proportions. In another published study, treatment discontinuation among RA patients reflected more the individual patient beliefs regarding treatment necessity and safety than the actual disease activity or route of drug administration [16]. In a second study, about half of RA patients prescribed methotrexate interrupted their treatment within 1 year; these patients had higher disease activity compared to those who remained on treatment [17]. We also cannot rule out the possibility that anti-TNF use increased complication awareness which may have prompted physician contacts at the onset of symptoms and prevented deterioration requiring hospitalizations.

Few studies have considered admission to hospital as an effectiveness indicator of anti-TNF and DMARDs use. An Israeli study reported a decreased frequency of the number of all-cause hospitalizations during anti-TNF treatment compared to the period before treatment among patients with RA and spondyloarthropathies (44.2 versus 74.2 hospitalizations/100 py, p-value < 0.0001). The authors reported similar tendency related to hospitalizations due to exacerbation of the rheumatic disease (21.9 versus 47.5/100 py, p <0.0001) and for orthopedic and surgical indications, however the latter analyses did not reach statistical significance [9]. A Japanese study reported no significant yearly difference in the prevalence of RA-related surgery after the introduction of anti-TNF drugs between 2004 and 2007, however a significantly higher proportion was observed for specific orthopedic surgery, starting in the second year. Among anti-TNF users, patients who had undergone RA-related surgery presented longer disease duration and higher functional disability compared to those who had not undergone this procedure [8]. Both of these studies were conducted shortly after the approval of anti-TNF drugs for the treatment of RA in their respective countries.

In our study, the decreasing trend of MSD-related hospitalizations found among DMARDs users over the three periods compared to the non-users perhaps, reflects the migration of the more severe cases from the DMARDs group after the approval of anti-TNF for reimbursement and over time.

Our study assessed the risk of MSD-related hospitalizations only. Hospitalizations for other reasons (including infections) are also important outcomes in RA patients, but were not within our study aim and were not investigated. Confounding by indication can greatly hinder the results of observational studies, because of the non-randomized nature of patient allocation to study treatments. The use of the propensity score methodology has been proposed to address this bias [18]. In our study, we used the high-dimensional propensity score to match patients in the treatment groups and create more balanced exposed and non-exposed groups [10]. In addition, we addressed selection bias in the design by constructing separate cohorts in two post-periods that separated the years immediately after the launch of the anti-TNF drugs (2002–2006) where channeling of more severe RA cases to anti-TNF treatment was very strong, from later years (2007–2011) where channeling remained but may have been less severe than that of earlier years [3, 5, 19–21].

## Conclusion

In conclusion, the results of this population-based study suggest that the risk of MSD-related hospitalizations is lower in patients using DMARD alone versus those not using DMARD or anti-TNF therapy. The risk of MSD-related hospitalizations in those in whom DMARDs only has failed and are put on anti-TNF (with or without DMARDs) was similar to that of patients not using either of these therapies. Similar risks in patients not using DMARD or anti-TNF treatment to that of those using anti-TNF, presumably the more severe, indicate that some RA patients requiring treatment are not using one and are at risk of RA complications and perhaps disability.

## Abbreviations

CHF, congestive heart failure ; CVD, cerebrovascular disease; DMARDs, disease modifying antirheumatic drugs ; ICD, Régie de l’assurance maladie du Québec ; MSD, musculoskeletal ; NSAIDs, Régie de l’assurance maladie du Québec ; Py, patient-years; RA, Rheumatoid arthritis ; RAMQ, Régie de l’assurance maladie du Québec; RCT, musculoskeletal ; SD, standard deviation ; SES, socioeconomic status ; SSRI, serotonin reuptake inhibitors ; TNF, Tumor Necrosis Factor

## Appendix

### Appendix 1

Frequency of MSD disorders coded as principal diagnoses among study individuals hospitalized for MSD conditions.

**Table 6:** Frequency of MSD disorders coded as principal diagnoses among study individuals hospitalized for MSD conditions

	2002–2006	2007–2011
	All treatment groups	All treatment groups
Total number of patients hospitalized for MSD conditions	155	145
Diagnoses	*N* (%)	
Rheumatoid arthritis (ICD-9: 714; ICD-10: M05, M06)	67 (43.23)	37 (25.52)
Osteoarthrosis and allied disorders (ICD-9: 715; ICD-10: M16- M19)	21 (13.55)	51 (35.17)
Acquired deformities of joints (ICD-9:717, 730, 733, 735, 736; ICD-10: M20-M24, M80, M84-87)	29 (18.72)	26 (17.93)
Other and unspecified disorders of back (ICD-9: 720–722, 724; ICD-10: M47, M48, M54)	19 (12.27)	8 (5.52)
Other disorders of synovium, tendon, and bursa (ICD-9: 726, 727, 728; ICD-10: M62, M65, M66, M70, M71, 75)	9 (5.82)	17 (8.35)
Other: ICD-9: 710, 716, 719, 729 ICD-10: M00, M13, M60, M72,M99	10 (6.46)	6 (4.14)

### Appendix 2

Frequency of the primary diagnoses of MSD hospitalizations during the period 2007–2011 (ICD-10 codes, as recorded in the database, are listed in alphabetical order).

**Table 7:** Frequency of the primary diagnoses of MSD hospitalizations during the period 2007–2011 (ICD-10 codes, as recorded in the database, are listed in alphabetical order)

	*N* (%)
Total number of patients hospitalized for MSD conditions during 2007–2011	145
M0097; pyogenic arthritis, unspecified, ankle and foot	1 (0.7)
M051, M053, M059 (rheumatoid lung disease *n* = 1; rheumatoid arthritis with involvement of other organs *n* = 3; seropositive rheumatoid arthritis, unspecified site *n* = 2)	6 (4.2)
M063, M068, M069 (rheumatoid nodule; *n* = 6; other specified rheumatoid arthritis, *n* = 1; rheumatoid arthritis, unspecified; *n* = 24)	31 (21.4)
M1315, M1397 (monoarthritis, pelvic region and thigh, *n* = 1; arthritis, unspecified, ankle and foot, *n* = 1)	2 (1.4
M167, M169 (coxarthrosis, secondary, *n* = 2; unspecified, *n* = 9)	11 (7.6)
M171, M179 (gonarthrosis, primary *n* = 5; unspecified, *n* = 28)	33 (22.8)
M189; arthrosis of first carpometacarpal joint, unspecified	2 (1.4)
M190, M199 (arthrosis, primary of other joints, *n* = 2; unspecified, *n* = 3)	5 (3.5)
M201, M202, M204 (hallux valgus, *n* = 9; hallux rigidus, *n* = 2; other hammer toe(s) (acquired), *n* = 1)	12 (8.3)
M2183; other specified acquired deformities of limbs, forearm	1 (0.7)
M224; chondromalacia patellae	1 (0.7)
M2320; derangement of meniscus due to old tear or injury, multiple sites	1 (0.7)
M2411; other articular cartilage disorders, shoulder region	1 (0.7)
M2587; other specified joint disorders, ankle and foot	1 (0.7)
M313; Wegener's granulomatosis	2 (1.4)
M4802, M4806, M4856 (Spinal stenosis, cervical region, *n* = 1; lumbar region, *n* = 3; collapsed vertebra, lumbar region, *n* = 1)	5 (3.5)
M545; low back pain	3 (2.1)
M6096; myositis, unspecified, lower leg	1 (0.7)
M6227, M6289 (ischaemic infarction of muscle, ankle and foot, *n* = 1; other specified disorders of muscle, unspecified site, *n* = 1)	2 (1.4)
M6593, M6289, M6596 (synovitis and tenosynovitis, forearm, *n* = 1; hand, *n* = 1; lower leg, *n* = 1)	3 (2.1)
M6657; spontaneous rupture of unspecified tendon, ankle and foot	1 (0.7)
M702; olecranon bursitis	2 (1.4)
M703; other bursitis of elbow	1 (0.7)
M7134, M7136, M7192 (other bursal cyst, hand, *n* = 2; lower leg, *n* = 1; bursopathy, unspecified, upper arm, *n* = 1)	4 (2.8)
M7263, M7192 (necrotizing fasciitis, forearm, *n* = 1, pelvic region and thigh, *n* = 1)	2 (1.4)
M751, M758 (rotator cuff syndrome, *n* = 1; other shoulder lesions, *n* = 1)	2 (1.4)
M8098; unspecified osteoporosis with pathological fracture, other site	1 (0.7)
M8448; pathological fracture, not elsewhere classified, other site	2 (1.4)
M8558; aneurysmal bone cyst, other site	1 (0.7)
M8617, M8697 (osteomyelitis ankle and foot, acute *n* = 1, unspecified *n* = 3)	4 (2.8)
M8795; osteonecrosis, unspecified, pelvic region and thigh	1 (0.7)

### Appendix 3

Use of NSAIDs and/or corticosteroids in follow-up. Descriptions of data: Frequency of MSD disorders coded as principal diagnoses among study individuals hospitalized for MSD conditions. Frequency of the primary diagnoses of MSD hospitalizations during the period 2007–2011 (ICD-10 codes, as recorded in the database, are listed in alphabetical order). Use of NSAIDs and/or corticosteroids in follow-up

**Table 8:** Use of NSAIDs and/or corticosteroids in follow-up

	Anti-TNF	DMARDs	Non- users
	2002–2006		
Total number of patients	557	1144	656
NSAIDs in follow-up	372 (66.8)	788 (68.9)	451 (68.8)
Corticosteroid in follow-up	344 (61.8)	650 (56.8)	353 (53.8)
NSAIDs and/or corticosteroid in follow-up	460 (82.6)	959 (84.7)	531 (80.9)
	2007–2012		
Total number of patients	690	1651	532
NSAIDs in follow-up	389 (56.4)	981 (59.4)	257 (48.3)
Corticosteroid in follow-up	413 (59.9)	790 (47.8)	276 (51.9)
NSAIDs and/or corticosteroid in follow-up	568 (82.3)	1251 (75.8)	396 (74.4)
